# Genome-wide identification of the *BCAT* gene family in *Zygophyllum brachypterum* and functional characterization of *ZbBCAT2* in salt stress response

**DOI:** 10.3389/fpls.2026.1978750

**Published:** 2026-10-19

**Authors:** Donghui Miao, Qi Ning, Yidan Yang, Zhibin Cui, Iqra Fiaz, Wenjuan Huang, Peipei Jiao

**Affiliations:** 1 Xinjiang Production and Construction Corps Key Laboratory of Protection and Utilization of Biological Resources in Tarim Basin, College of Life Science, Tarim University, Alar, China; 2 College of Horticulture and Forestry, Tarim University, Alar, Xinjiang, China

**Keywords:** branched-chain amino acid transaminase, functional characterization, salt stress, *Zygophyllum brachypterum*, gene family

## Abstract

Soil salinization is a major abiotic stress limiting plant growth and stress adaptation. Branched-chain amino acid aminotransferases (BCATs) govern branched-chain amino acid (BCAA) metabolic homeostasis and are closely associated with plant abiotic stress tolerance, yet their genome-wide characteristics and functions in the desert halophyte *Zygophyllum brachypterum* remain unknown. In this study, five *ZbBCAT* genes were identified at the genome-wide level and systematically characterized via phylogenetic, syntenic, structural and promoter cis-element analyses; their spatiotemporal expression profiles under salt stress were determined by qRT-PCR, and the function of the core candidate *ZbBCAT2* (*Zb8G000817*) was verified via heterologous overexpression in *Arabidopsis thaliana* and *Oryza sativa*. The results showed that the five *ZbBCAT* members clustered into three evolutionary subfamilies with marked monocot–dicot divergence; *ZbBCAT2* was the only member consistently and strongly induced by salt stress across roots, stems and leaves with the highest upregulation amplitude; and *ZbBCAT2* overexpression exacerbated salt stress damage and reduced survival rates in both *A. thaliana* and *O. sativa*, while the *bcat2* mutant exhibited enhanced tolerance, revealing that *ZbBCAT2* exerts consistent negative regulatory effects on salt tolerance when heterologously expressed in both dicot and monocot plant systems. This study provides systematic insights into the *ZbBCAT* family and identifies *ZbBCAT2* as a key negative regulator of salt tolerance, offering candidate genes and mechanistic references for plant salt tolerance improvement.

## 1 Introduction

Soil salinization is a major abiotic stress factor that restricts plant growth, development, and the distribution of natural vegetation. Under salt stress, plants accumulate a range of protective metabolites such as amino acids, flavonoids, and gibberellins to mitigate stress-induced damage (Chen et al., 2022). Nitrogen (N) is an essential macronutrient for plant growth and development (Näsholm et al., 2009), and amino acids serve as the primary form of nitrogen transport and storage in most plant species (Tegeder and Rentsch, 2010). As precursors and fundamental building blocks of proteins (Hayat et al., 2012), amino acids play a central role in plant growth and developmental processes. To meet the nitrogen demands of different organs, amino acids are transported and allocated between source and sink tissues such as roots and leaves via the vascular system, ensuring efficient nitrogen utilization (Nie et al., 2025). Meanwhile, amino acids also act as signaling molecules involved in regulating plant growth, development, and responses to environmental stresses.

Valine, leucine, and isoleucine are collectively termed branched-chain amino acids (BCAAs) due to their shared short branched alkyl side chains (Galili et al., 2016). These amino acids can be synthesized *de novo* in plants but cannot be produced autonomously in humans, making them essential dietary amino acids. Extensive studies have demonstrated that amino acid accumulation is closely associated with plant stress tolerance (Taylor et al., 2004; Hayat et al., 2012). Amino acids and their metabolites function as compatible osmolytes to enhance plant resilience to adverse environments (Campalans et al., 1999; Ashraf and Foolad, 2007; Joshi et al., 2010). For instance, stress-induced proline accumulation has been documented not only in plants but also in eubacteria, protozoa, and algae (Maggio et al., 2002), and its accumulation level is positively correlated with stress tolerance (Lehmann et al., 2010).Proline is not the only amino acid that accumulates in plants under abiotic stress. Research shows that BCAA content can increase by several folds under osmotic stress (Joshi et al., 2010). Similar to proline, BCAAs may participate in the regulation of plant salt tolerance by acting as compatible osmolytes or alternative energy substrates, maintaining cell turgor under water deficit, and alleviating the rapid decline of leaf water potential (Wani et al., 2013).

Branched-chain amino acid aminotransferases (BCATs) are key regulatory enzymes in branched-chain amino acid metabolism (Xing and Last, 2017). The transamination reaction catalyzed by BCATs constitutes both the final step of BCAA biosynthesis and the first step of BCAA catabolism. In the biosynthetic pathway, BCATs catalyze the conversion of 4-methyl-2-oxopentanoate to leucine, 3-methyl-2-oxobutanoate to valine, and 3-methyl-2-oxopentanoate to isoleucine, respectively (Schuster and Binder, 2005). To date, the *BCAT* gene family has been identified at the genome-wide scale in various plant species, including *Arabidopsis thaliana* (Diebold et al., 2002; Schuster and Binder, 2005; Schuster et al., 2006; Binder, 2010), *Oryza sativa* (Malatrasi et al., 2006), *Solanum tuberosum* (Gao et al., 2009), *Solanum lycopersicum* (Gonda et al., 2010; Maloney et al., 2010), and *Malus domestica* (Yang et al., 2026).Owing to their stress-inducible expression profiles and close correlation with BCAA content dynamics, BCATs are regarded as key modulators of BCAA metabolic homeostasis in plants under stress conditions (Urano et al., 2009; Joshi et al., 2010; Chen et al., 2023). For example, *AtBCAT2* has been confirmed to mediate abscisic acid (ABA)-dependent BCAA accumulation in *A. thaliana* (Urano et al., 2009), and both *AtBCAT1* and *AtBCAT2* are significantly upregulated under salt stress (Joshi et al., 2010). In *O. sativa*, *OsBCAT2* modulates plant salt tolerance by regulating the vitamin B5 biosynthetic pathway (Sun et al., 2024). In coordination with *BCAT* functions, transcript levels of key catabolic genes such as *BCKD* and *IVD* also increase under stress (Peng et al., 2015). These findings indicate that BCATs support energy metabolism and signal transduction in plants under salt stress by regulating the dynamic metabolic balance of BCAAs. Nevertheless, the molecular regulatory network underlying stress-induced BCAA accumulation in plants and the precise functional mechanisms of BCATs in stress responses remain to be systematically elucidated.

*Zygophyllum brachypterum* is a typical desert-adapted species with strong abiotic stress tolerance. However, the genome-wide characterization of the *BCAT* gene family, its regulatory role in BCAA accumulation, and its functional mechanisms underlying salt stress response remain largely uncharacterized in this species. *Z. brachypterum*, a perennial herb or subshrub of the genus *Zygophyllum*, is a dominant stress-tolerant species in desert and semi-desert ecosystems. It possesses ecological restoration, medicinal, and forage values, and plays a critical role in vegetation restoration and stress-resistant germplasm exploitation in arid regions. In this study, we performed genome-wide identification and bioinformatics characterization of the *BCAT* gene family in *Z. brachypterum*, and analyzed the spatiotemporal expression patterns of family members under salt stress via qRT-PCR. On this basis, we cloned the core gene *ZbBCAT2*(*Zb8G000817*) and verified its function in salt stress response via heterologous transformation in *A. thaliana* and *O. sativa*. Our findings lay a theoretical foundation for elucidating the molecular mechanism by which *ZbBCAT* genes regulate BCAA accumulation in *Z. brachypterum*, and provide candidate targets for genetic improvement of plant stress tolerance.

## 2 Materials and methods

### 2.1 Genome-wide identification of the *BCAT* gene family in *Z. brachypterum*

The whole-genome sequence, coding sequences (CDSs), and protein sequences of *Z. brachypterum* were obtained from our laboratory’s prior *de novo* whole-genome sequencing, assembly and structural annotation work. *A. thaliana* BCAT protein sequences were downloaded from TAIR (https://www.arabidopsis.org/) as queries.

TBtools-II v2.119 was used for candidate screening. First, BLASTP alignment was performed against the *Z. brachypterum* proteome with an E-value cutoff of 1e-5. Separately, the HMM profile of Aminotran_4 (PF01063) was downloaded from InterPro (https://www.ebi.ac.uk/interpro/) and used for HMM-based screening.

Redundant sequences from the two approaches were removed, and remaining candidates were validated for intact conserved domains via NCBI CDD (https://www.ncbi.nlm.nih.gov/cdd/) and SMART database (https://smart.embl.de/), with incomplete sequences discarded. Reciprocal BLASTP analysis against the *A. thaliana* proteome was performed using TBtools-II v2.119 to confirm homology with known AtBCATs, yielding the final *ZbBCATs* gene set.

### 2.2 Physicochemical property and subcellular localization prediction

Basic physicochemical parameters (amino acid length, molecular weight, pI, instability index, aliphatic index, GRAVY) were predicted using ExPASy ProtParam (https://web.expasy.org/protparam/). Subcellular localization was predicted with WOLF PSORT (https://wolfpsort.hgc.jp/).

### 2.3 Chromosomal localization analysis

Chromosomal distribution analysis was conducted using TBtools-II v2.119, with the whole-genome FASTA sequence and GFF3 structural annotation file of *Z. brachypterum* as input data sources. Specifically, the Fasta Statistics module was applied to calculate chromosome lengths from the genomic sequence file; the GFF3 Gene Position Parsing module was used to extract the exact chromosomal coordinates of all *ZbBCAT* genes from the annotation file; and the Gene Position Visualization tool was employed to generate and visualize the final chromosomal location map of the *ZbBCAT* gene family.

### 2.4 Phylogenetic analysis

First, full-length BCAT protein sequences of *A. thaliana*, *P. euphratica*, *O. sativa*, and *Z. mays* were retrieved from public databases: *A. thaliana* sequences from the Arabidopsis Information Resource (TAIR, https://www.arabidopsis.org/), and sequences of *O. sativa*, *Z. mays* and *P. euphratica* from the NCBI Protein database (https://www.ncbi.nlm.nih.gov/protein/). Homologous gene pairs between *Z. brachypterum* and each reference species were identified via BLASTP implemented in TBtools-II v2.119, set with an E-value cutoff of 1e-5 and using the reciprocal best hit strategy to confirm orthologous relationships. Second, all validated full-length BCAT protein sequences were subjected to multiple sequence alignment using the MUSCLE algorithm integrated in MEGA 12, with default gap opening and gap extension penalties. Third, the optimal amino acid substitution model for phylogenetic reconstruction was selected via the Model Selection function in MEGA 12. Based on the lowest Bayesian Information Criterion (BIC) value, the WAG+G+I model was determined as the best-fit model, which incorporates the Whelan and Goldman (WAG) amino acid substitution matrix, gamma-distributed rate heterogeneity across sites (G), and a proportion of invariant sites (I). Fourth, a ML phylogenetic tree was constructed using the selected WAG+G+I model. Gaps and missing data in the alignment were treated using the pairwise deletion option to retain maximum valid sequence information. The reliability of each internal branch was evaluated via bootstrap analysis with 1000 replicate samplings. The resulting phylogenetic tree was annotated and exported for subsequent visualization and interpretation.

### 2.5 Multi-species synteny and gene duplication analysis

Genome-wide synteny analysis was conducted between *Z. brachypterum* and each of the four reference species. Homologous gene pairs were identified via BLASTP, syntenic blocks were detected with the MCScanX module in TBtools-II v2.119, and BCAT-associated synteny relationships were extracted for visualization.

### 2.6 Gene structure and conserved domain analysis

Conserved motifs were identified with MEME v5.4.1 (http://meme-suite.org), set to a minimum width of 10 aa, maximum width of 150 aa, and maximum of 10 motifs. Functional domains were annotated with Pfam (https://ngdc.cncb.ac.cn/databasecommons/database/id/186) and NCBI CDD. Exon–intron structures were extracted from the GFF3 file and plotted with TBtools-II v2.119.

### 2.7 Promoter cis-acting element analysis

The 2000 bp sequences upstream of each *ZbBCAT* translation start site were extracted. Cis-acting elements were predicted using PlantCARE (https://bioinformatics.psb.ugent.be/webtools/plantcare/html) and visualized with TBtools-II v2.119.

### 2.8 Protein secondary and tertiary structure prediction

Secondary structure was predicted with SOPMA (https://npsa.lyon.inserm.fr/cgi-bin/npsa_automat.pl?page=/NPSA/npsa_sopma.html). For each ZbBCAT protein, the proportions of α-helix, β-turn, extended strand, and random coil were calculated as the ratio of the number of amino acid residues assigned to each structural category by the SOPMA algorithm to the total number of residues in the full-length protein sequence. Tertiary structure homology modeling was performed with SWISS-MODEL (https://swissmodel.expasy.org/interactive), using the highest-homology template and outputting quality-evaluated 3D models.

### 2.9 Plant materials and stress treatments

Seeds of *Z. brachypterum* (a wild desert plant species with no formally established cultivars) were collected from Tielieke Town, Baicheng County, Aksu, Xinjiang, China (81°36′31.5″ E, 41°52′2.6″ N, 1522.7 m a.s.l.). Seeds were surface-sterilized with 20% NaClO for 15 min, rinsed 5–6 times with sterile water, plated on 7 g/L agarose medium, and germinated in the dark at 25 °C for 2 d.

Germinated seedlings were transplanted to a vermiculite:perlite:nutrient 3:3:1 (v/v/v) soil mixture and grown in a chamber under 16 h light/8 h dark, 25 °C, 60% relative humidity. At the 10-true-leaf stage, plants were treated with 500 mM NaCl. Roots, stems, and leaves were harvested at 0, 24, 48, and 72 h, snap-frozen in liquid nitrogen, and stored at −80 °C. Three biological replicates were included per time point.

*A. thaliana* materials were Col-0 wild type and *bcat2* T-DNA insertion mutant, grown at 22 °C, 16 h light/8 h dark, 60% RH. *O. sativa* material was cv. Zhonghua 11 (ZH11), grown at 28 °C, 16 h light/8 h dark, 70% RH. All plants were watered regularly.

### 2.10 Total RNA extraction and qRT-PCR analysis

Tissues were ground to powder in liquid nitrogen. Approximately 100 mg of snap-frozen plant tissue was ground to a fine powder in liquid nitrogen using a pre-cooled mortar and pestle, and immediately homogenized in 1 mL of RNAisol reagent (Simgen, Cat. No. 5301100, Hangzhou, China) by thorough vortexing. The homogenate was incubated at room temperature for 5 min to allow complete dissociation of nucleoprotein complexes.For phase separation, 200 μL of chloroform was added per 1 mL of RNAisol reagent. The tube was vigorously vortexed for 15 s and incubated at room temperature for 3 min, followed by centrifugation at 12,000 × g for 15 min at 4 °C. The upper colorless aqueous phase (containing total RNA) was carefully transferred to a new RNase-free centrifuge tube, with care taken to avoid disturbing the middle interphase and lower organic phase.

For RNA precipitation, an equal volume of isopropanol was added to the aqueous phase, mixed gently by inverting the tube, and incubated at room temperature for 10 min. The mixture was centrifuged at 12,000 × g for 10 min at 4 °C to pellet the RNA, after which the supernatant was completely discarded. The RNA pellet was washed twice with 1 mL of 75% DEPC-treated ethanol. For each wash, the pellet was fully resuspended by vortexing, then centrifuged at 7,500 × g for 5 min at 4 °C. After removing the supernatant, residual ethanol was eliminated by a short spin and careful pipette aspiration, and the pellet was air-dried at room temperature for 3–5 min. Finally, the purified RNA was resuspended in 30–50 μL of DEPC-treated water and fully dissolved by incubation at 55 °C for 5 min. RNA concentration and purity were determined by spectrophotometric measurement of A260/A280 ratio, and RNA integrity was verified by 1.2% agarose gel electrophoresis. RNA concentration and purity were measured with an ultramicro spectrophotometer (Kaiao, K5800, Beijing, China). RNA concentration was quantified by absorbance at 260 nm, and purity was evaluated by the A260/A280 and A260/A230 absorbance ratios.

1 µg of total RNA was reverse-transcribed into first-strand cDNA using the SPARKscript II RT Plus Kit (Sparkjade, Cat. No. AG0304, Shandong, China) according to the manufacturer’s instructions. qRT-PCR was performed on a LineGene 9600 fluorescent quantitative PCR detection system (Bioer, Hangzhou, China) with gene-specific primers in a 10 μL reaction system, which contained 5 μL 2× Universal SYBR Green qPCR Mix (Sparkjade, Cat. No. A0105, Shandong, China), 0.2 μL each of forward and reverse primers, 1.5 μL cDNA template, and 3.1 μL nuclease-free ddH_2_O. The thermal cycling program was set as follows: initial denaturation at 95 °C for 30 s; 45 cycles of denaturation at 95 °C for 10 s, annealing at 58 °C for 10 s, and extension at 72 °C for 30 s; followed by standard melting curve analysis to confirm primer specificity.

The amplification efficiency of each primer pair was determined via 5-point serial dilution of cDNA templates. As shown in **Supplementary Table S1**, all primer efficiencies ranged from 93.7% to 99.3% with R² > 0.98, satisfying the quantitative performance standard. *ZbActin*, *AtActin* and *OsActin* were used as endogenous reference genes for *Z. brachypterum*, *A. thaliana* and *O. sativa*, respectively. All reference genes exhibited highly stable expression across all experimental conditions, with the coefficient of variation of Ct values below 3% across all samples. For each experimental treatment, three independent biological replicates were prepared using separately cultured and treated plant materials, and each biological sample was assayed in three technical replicates. Relative gene expression levels were calculated using the 2^^-ΔΔCt^ method (Livak and Schmittgen, 2001).

### 2.11 Vector construction and plant genetic transformation

#### 2.11.1 Construction of *ZbBCAT2* overexpression vector and transformation into *Agrobacterium tumefaciens*

The pGreen-YFP vector, harboring a CaMV 35S promoter and a C-terminal YFP reporter tag, was adopted as the backbone for overexpression construct generation. To preserve the intact N-terminal chloroplast transit peptide and central catalytic domain of *ZbBCAT2* (*Zb8G000817*), the CDS of *ZbBCAT2* without the termination codon was fused upstream of the YFP sequence to produce a C-terminal YFP fusion protein. A 35S::YFP empty vector without the target gene insert was constructed in parallel and served as the negative control for all overexpression experiments. Primers carrying *Hind III* sites and 15 bp homology arms matching the pGreen-YFP vector were designed for the full-length *ZbBCAT2* CDS. PCR was performed with 2×Phanta Max Super-Fidelity DNA Polymerase (Vazyme, Cat. No. P505, Nanjing, China) using leaf cDNA as template: 95 °C 3 min; 30 cycles of 95 °C 15 s, 58 °C 15 s, 72 °C 1 min; 72 °C 1 min final extension.

PCR products were separated on 0.8% agarose gels and purified with a gel extraction kit (TIANGEN, Cat. No. DP209, Beijing, China). pGreen-YFP was linearized with *Hind III* at 37 °C for 4 h, purified, and ligated with the insert at a 1:2 molar ratio using 2× MultiF Seamless Assembly Mix (ABclonal, Cat. No. RK21020, Wuhan, China) at 50 °C for 15 min, yielding the 35S::*ZbBCAT2*-YFP overexpression construct.

Ligation products were transformed into chemically competent *Escherichia coli* DH5α cells (Dalin Bio, Cat. No. DLC114, Beijing, China), plated on LB + 50 mg/L kanamycin, and incubated at 37 °C for 12–16 h. Positive clones were verified by colony PCR and Sanger sequencing. Correct plasmids were transformed into *Agrobacterium tumefaciens* GV3101 competent cells (Angyu Bio, Cat. No. G6070, Shanghai, China) via the freeze-thaw method. Transformants were selected on LB + 50 mg/L rifampicin + 50 mg/L kanamycin at 28 °C for 2–3 d, verified by PCR, and stored as 20% glycerol stocks at −80 °C.

#### 2.11.2 Subcellular localization analysis

For subcellular localization analysis, the glycerol stocks of *A. tumefaciens* GV3101 strains carrying 35S::YFP and 35S::*ZbBCAT2*-YFP constructs were streaked on LB plates supplemented with 50 mg/L rifampicin and 50 mg/L kanamycin, and incubated at 28 °C for 2 d. Single colonies were inoculated into liquid LB medium with the same antibiotics and cultured overnight at 28 °C with shaking at 200 rpm. The bacterial cells were harvested by centrifugation, resuspended in infiltration buffer (10 mM MES, 10 mM MgCl_2_, 200 μM acetosyringone, pH 5.6), adjusted to an OD_600_ of 0.6–0.8, and incubated at room temperature in the dark for 2–3 h.

The bacterial suspensions were then infiltrated into the abaxial epidermis of fully expanded leaves from 4-week-old *Nicotiana benthamiana* seedlings. After 48–72 h of incubation under normal growth conditions, the infiltrated leaf regions were excised and mounted on glass slides for microscopic observation. Fluorescence signals were detected using a laser scanning confocal microscope. The detection parameters were set as follows: for YFP fluorescence, excitation wavelength at 488 nm and emission wavelength at 527 nm; for chlorophyll autofluorescence, excitation wavelength at 488 nm and emission wavelength at 681 nm, with a detection bandwidth of 10 nm. Bright field images were acquired simultaneously to show cell morphology, and merged images of the three channels were generated to determine the subcellular localization pattern of ZbBCAT2 protein.

#### 2.11.3 *A. thaliana* transformation and homozygous line screening

Transformation was performed via the floral dip method. Agrobacterium was grown to OD_600_ ≈ 0.6, pelleted, and resuspended in infiltration buffer (1/2 MS, 5% sucrose, 0.02% Silwet L-77, pH 5.8) to OD_600_ = 0.6. Inflorescences were dipped for 30 s, and plants were kept dark and moist for 24 h before return to normal growth. T_0_ seeds were harvested from individual plants.

T_0_ seeds were sterilized (75% ethanol 30 s, 20% NaClO 10 min), sown on 1/2 MS + 50 mg/L hygromycin, vernalized at 4 °C for 3 d, and grown in the chamber. Resistant seedlings were transplanted, genotyped by genomic PCR, and selfed to T_3_ generation. The empty vector control lines were generated and screened following exactly the same transformation and breeding procedure. The expression levels of *ZbBCAT2* overexpression lines were confirmed by qRT-PCR, and three independent homozygous lines (OE6, OE27, OE8) were selected for subsequent phenotyping assays.

The *bcat2* T-DNA insertion mutant (SALK_037854) in the Col-0 background was obtained from the SALK mutant collection. The T-DNA is located in the 5th exon of *AtBCAT2* (*AT3G19710*). Homozygous lines were screened by three-primer genotyping with LP, RP and LBb1.3 primers, and the null expression of endogenous *AtBCAT2* was confirmed by qRT-PCR before subsequent experiments.

Wild-type Col-0, *bcat2* loss-of-function mutant, empty vector control lines, and the three *ZbBCAT2* overexpression lines were grown under identical environmental conditions for all experiments. All phenotypic and physiological indicators were compared with the empty vector control to exclude non-specific effects caused by the transformation procedure, vector backbone or YFP tag.

#### 2.11.4 *O. sativa* transformation and homozygous line screening

ZH11 was transformed via Agrobacterium-mediated callus transformation. Dehulled mature seeds were sterilized, plated on callus induction medium, and grown in the dark at 28 °C. Embryogenic calli were immersed in Agrobacterium suspension (OD_600_ ≈ 0.6, 1/2 MS medium + 100 μmol/L acetosyringone) for 15 min, blotted dry, and co-cultivated on filter paper-laid medium at 26 °C for 3 d in the dark.

Calli were washed with 500 mg/L cefotaxime, blotted dry, and selected on medium with antibiotics and cefotaxime for two 2-week rounds at 28 °C dark. Resistant calli were transferred to differentiation medium under 16 h light/8 h dark at 28 °C. Regenerated plantlets were rooted, acclimatized, and transplanted to soil. T_0_ plants were genotyped by PCR and qRT-PCR, then selfed to T_2_ homozygous lines for phenotyping.

### 2.12 Salt stress treatment and phenotypic assays

#### 2.12.1 *A. thaliana* salt tolerance assay

Four-week-old seedlings of uniform size were transplanted to equal volumes of soil and treated with 500 mM NaCl every 3 d for 3 weeks. Leaf bleaching rate was calculated as: number of fully albino leaves/total leaf number × 100%. Three biological replicates (15 plants each) were used per genotype.

#### 2.12.2 *O. sativa* salt tolerance assay

Germinated seeds were sown in soil and grown to the two-leaf stage. Seedlings were treated with 500 mM NaCl every 2 d for 2 weeks. Survival rate was scored, with complete leaf yellowing and meristem necrosis defined as death. Three biological replicates (30 plants each) were used per genotype.

### 2.13 Statistical analysis

All experiments included three biological replicates. Quantitative data are presented as mean ± standard deviation (mean ± SD). Data processing and basic calculations were performed in Microsoft Excel 2019. Statistical analysis was conducted in SPSS 26.0, with an independent samples t-test used for two-group comparisons and two-way analysis of variance (two-way ANOVA) followed by Tukey’s *post-hoc* test for multi-group comparisons involving two experimental factors. A P-value less than 0.05 was considered statistically significant, and a P-value less than 0.01 was considered extremely significant. Figures were generated with GraphPad Prism 10.6.0 and TBtools-II v2.119.

## 3 Results

### 3.1 Identification of *ZbBCAT* gene family members in *Z. brachypterum*

Candidate genes were screened by BLAST search for homologous regions between *Z. brachypterum* and *A. thaliana*. Meanwhile, the Hidden Markov Model (HMM) of the BCAT characteristic domain Aminotran_4 (PF01063) was downloaded from the Pfam database, and conserved domain validation was performed via NCBI CD-search to retain proteins with intact functional domains. After removing redundant sequences and filtering for conserved domains using both self-constructed and downloaded HMM profiles, a total of 5 *ZbBCAT* gene family members were identified. The basic physicochemical properties of ZbBCAT proteins are summarized in **Table 1**. The encoded amino acid sequences range from 354 residues (Zb1G021424) to 555 residues (Zb1G011066), with molecular weights from 38 kDa (Zb1G021424) to 62 kDa (Zb1G011066). Theoretical isoelectric points (pI) range from 5.76 to 9.36: Zb8G000817 and Zb8G000819 have pI values above 7 and are alkaline proteins, while the remaining ZbBCAT proteins have pI values below 7 and are acidic proteins. Instability indices range from 41.77 (Zb8G000819) to 52.72 (Zb5G001696), all greater than 40, indicating that all ZbBCAT proteins are biochemically unstable (Guruprasad et al., 1990). All family members exhibit negative GRAVY values, confirming their hydrophilic nature. Subcellular localization prediction revealed distinct subcellular distribution patterns among ZbBCAT family members. Zb8G000817, Zb8G000819, and Zb5G001696 were predicted to be predominantly localized to chloroplasts, whereas Zb1G011066 and Zb1G021424 were predicted to localize mainly to the cytoplasm.

**Table 1:** Physicochemical properties and predicted subcellular localization of ZbBCAT family proteins in *Z. brachypterum*.

Sequence ID	Number of amino acid	Molecular weight (kDa)	Theoretical pI	Instability index	Aliphatic Index	Grand average of hydropathicity (GRAVY)	Prediction of subcellular localization
Zb8G000817	390	43.07	8.35	46.01	85.97	-0.225	chlo
Zb8G000819	414	45.86	9.36	41.77	91.84	-0.102	chlo
Zb5G001696	417	45.32	6.84	52.72	89.52	-0.044	chlo
Zb1G011066	555	62.61	6.39	45.06	90.43	-0.285	cyto
Zb1G021424	354	38.31	5.76	45.26	88.98	-0.067	cyto

### 3.2 Chromosomal Localization of *ZbBCAT* Gene Family Members

A chromosomal distribution map of *ZbBCAT* genes was constructed based on the *Z. brachypterum* genome assembly (**Figure 1**). Chromosomal mapping showed that family members are distributed on chromosomes 1, 5, and 8, with most located at chromosomal termini. *Zb8G000817* and *Zb8G000819* are closely linked and physically adjacent on chromosome 8, suggesting potential synergistic regulation or functional redundancy. Detailed genomic coordinates of all five *ZbBCAT* genes are summarized in **Supplementary Table S2**. To further elucidate the evolutionary selection pressure acting on this paralog pair, we calculated the non-synonymous substitution rate (Ka), synonymous substitution rate (Ks) and Ka/Ks ratio (**Supplementary Table S3**). The resulting Ka/Ks ratio was 0.454 (Ka = 0.099, Ks = 0.218), a value markedly lower than 1, demonstrating that both duplicated copies have been subject to strong purifying selection and have maintained functional constraints following the duplication event, rather than accumulating degenerative mutations. This evolutionary pattern supports the potential for synergistic regulation or functional redundancy between the two paralogs. The remaining three members are independently distributed on separate chromosomes, with no obvious tandem clustering.

**Figure 1:**
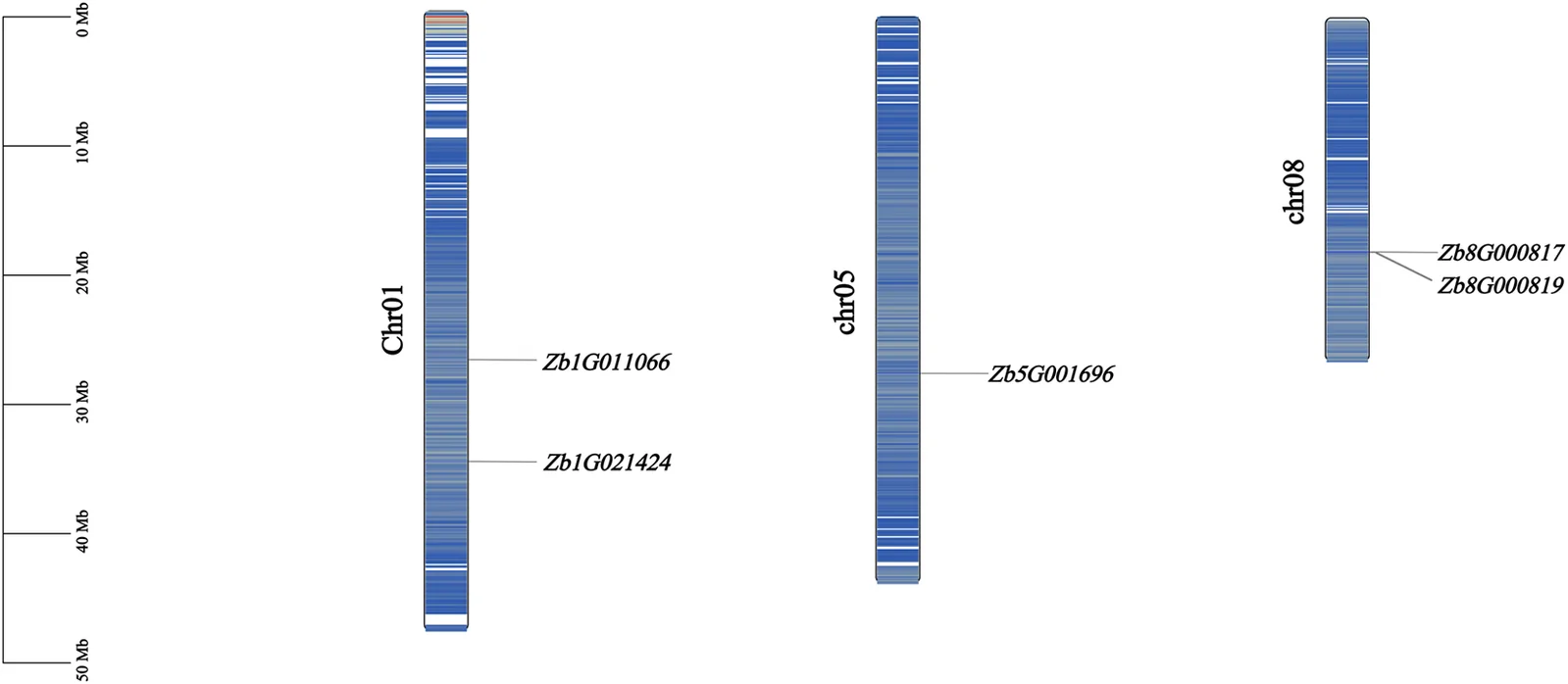
Chromosome location. Blue indicates low gene density on the chromosome, while red indicates high gene density on the chromosome.

### 3.3 Phylogenetic evolution analysis of *ZbBCAT* gene family members

To clarify the evolutionary status and phylogenetic relationships of the ZbBCAT family, full-length BCAT protein sequences from two dicot species (*A. thaliana* and *P. euphratica*) and two monocot species (*O. sativa* and *Z. mays*) were used to construct a ML phylogenetic tree (**Figure 2**). The tree was generated using the WAG+G+I amino acid substitution model with 1000 bootstrap replicates. All BCAT proteins clustered into three distinct evolutionary subfamilies, designated BCAT3, BCAT1/2, and BCAT4/5/6, with pronounced lineage-specific evolutionary patterns between monocot and dicot plants.

**Figure 2:**
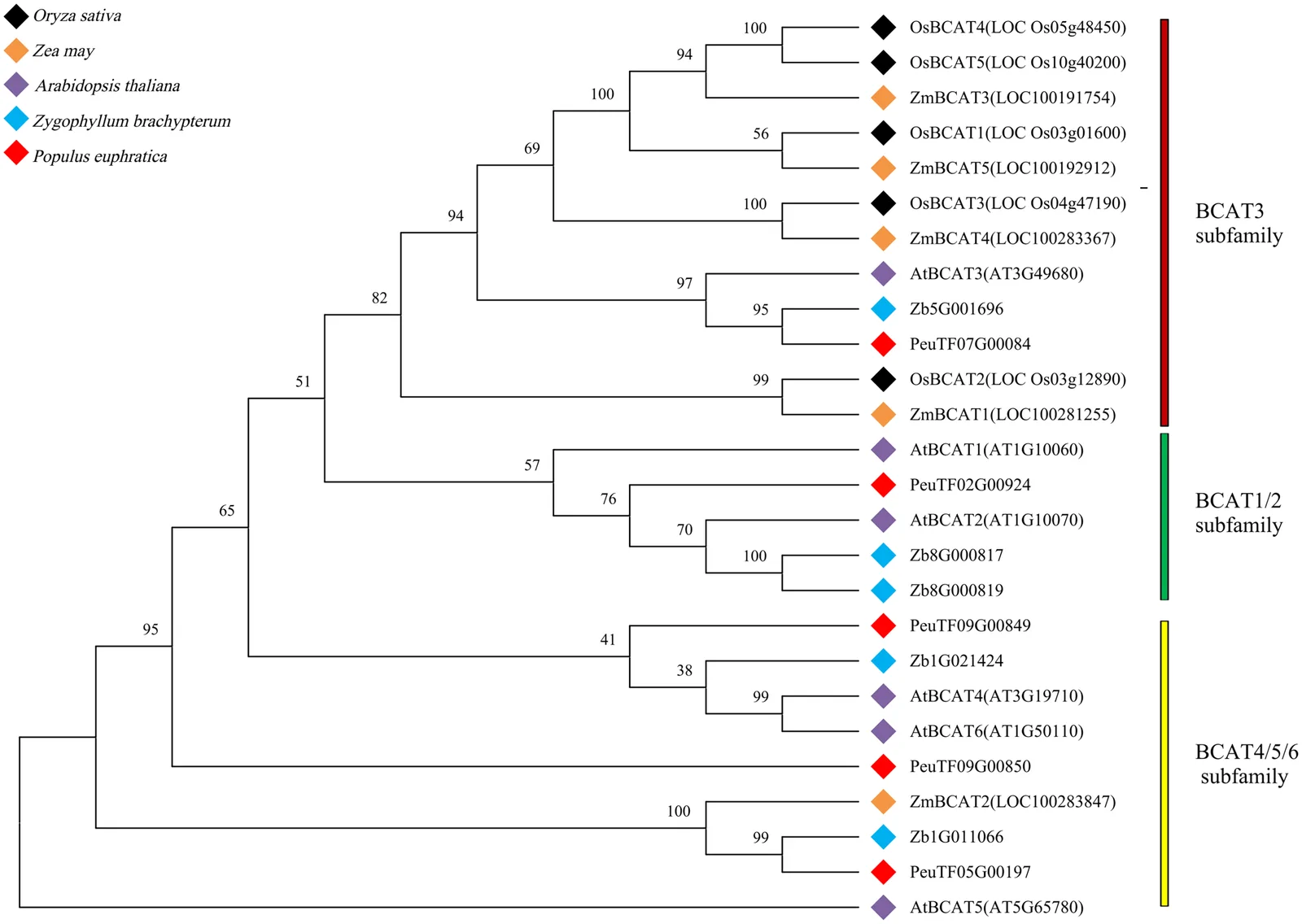
Phylogenetic analysis of the BCAT protein family from five representative plant species. A ML phylogenetic tree was constructed based on full-length BCAT amino acid sequences from *O. sativa*, *Z. mays*, *P. euphratica*, *Z. brachypterum* and *A. thaliana*. The tree was generated using the WAG+G+I amino acid substitution model with 1000 bootstrap replicates. Numbers at branch nodes indicate bootstrap support values. black for *O. sativa*, orange for *Z. mays*, red for *P. euphratica*, blue for *Z. brachypterum*, and purple for *A. thaliana*. Three distinct evolutionary subfamilies (BCAT3, BCAT1/2 and BCAT4/5/6) are labeled on the right side of the tree.

The BCAT3 subfamily formed the largest clade with generally high bootstrap support, and exhibited obvious copy number variation between the two plant lineages. Multiple BCAT members from *O. sativa* and *Z. mays* were assigned to this subfamily, reflecting gene expansion events that occurred during monocot evolution. By contrast, each dicot species harbored only one single-copy ortholog: AtBCAT3 (AT3G49680) in *A. thaliana*, PeuTF07G00084 in *P. euphratica*, and Zb5G001696 in *Z. brachypterum*. This copy number pattern indicates that the core metabolic function of BCAT3 is highly conserved in dicots, while the expanded copies in monocots may have partial functional redundancy.

The BCAT1/2 subfamily represented a dicot-specific evolutionary clade, with no orthologous members from *O. sativa* or *Z. mays* clustered into this group. Within this subfamily, Zb8G000817 and Zb8G000819 from *Z. brachypterum* formed a highly supported monophyletic branch (bootstrap = 100), indicating that the two paralogs originated from a species-specific gene duplication event. In comparison, *P. euphratica* retained only one homolog (PeuTF02G00924) in this subfamily, whereas *A. thaliana* possessed two paralogs (AtBCAT1 and AtBCAT2). This duplication pattern suggests that the two ZbBCAT paralogs may have undergone partial functional redundancy or subfunctionalization during the adaptive evolution of *Z. brachypterum* to harsh desert habitats.

The BCAT4/5/6 subfamily showed relatively deep evolutionary divergence, with low bootstrap support for some internal nodes, indicating high sequence differentiation among family members. Compared with the three copies (AtBCAT4, AtBCAT5, AtBCAT6) in *A. thaliana*, *Z. brachypterum* retained only two members (Zb1G011066 and Zb1G021424); similarly, *P. euphratica* and *Z. mays* also carried a reduced number of copies in this subfamily. This pattern suggests that gene contraction and functional specialization have occurred in the BCAT4/5/6 subfamily during the evolution of most plant lineages.

### 3.4 Synteny analysis of *BCAT* gene family members across multiple species

To elucidate the evolutionary origin and sequence conservation of the ZbBCAT family from a genomic perspective, genome-wide synteny alignments were performed between *Z. brachypterum* and two dicots (*A. thaliana*, *P. euphratica*) and two monocots (*O. sativa*, *Z. mays*), focusing on orthologous syntenic relationships of BCAT loci (**Figure 3**). Highly conserved synteny was observed among dicot species. Five pairs of BCAT syntenic homologs were identified between *Z. brachypterum* and *A. thaliana*, as well as between *Z. brachypterum* and *P. euphratica* (**Figures 3A, B**). Syntenic blocks cover all BCAT-bearing regions on chromosomes 1, 5, and 8 of *Z. brachypterum*, indicating that the BCAT family has retained an intact syntenic backbone during dicot evolution, with high conservation of core metabolic functions. In contrast, synteny between *Z. brachypterum* and the two monocot species was markedly reduced: only one pair of BCAT syntenic homologs was detected in each comparison, corresponding exclusively to the most conserved BCAT3 subfamily (**Figures 3C, D**). Chromosomal synteny of other family members was gradually lost during monocot–dicot divergence, indicating pronounced evolutionary and functional differentiation of the BCAT family between the two plant lineages. Against the genome-wide background of homologous synteny, the evolutionary pattern of the BCAT family aligns with the phylogenetic divergence of monocots and dicots, while also reflecting lineage-specific evolutionary trajectories. These findings provide direct genomic synteny evidence for the functional evolution of the plant BCAT family.

**Figure 3:**
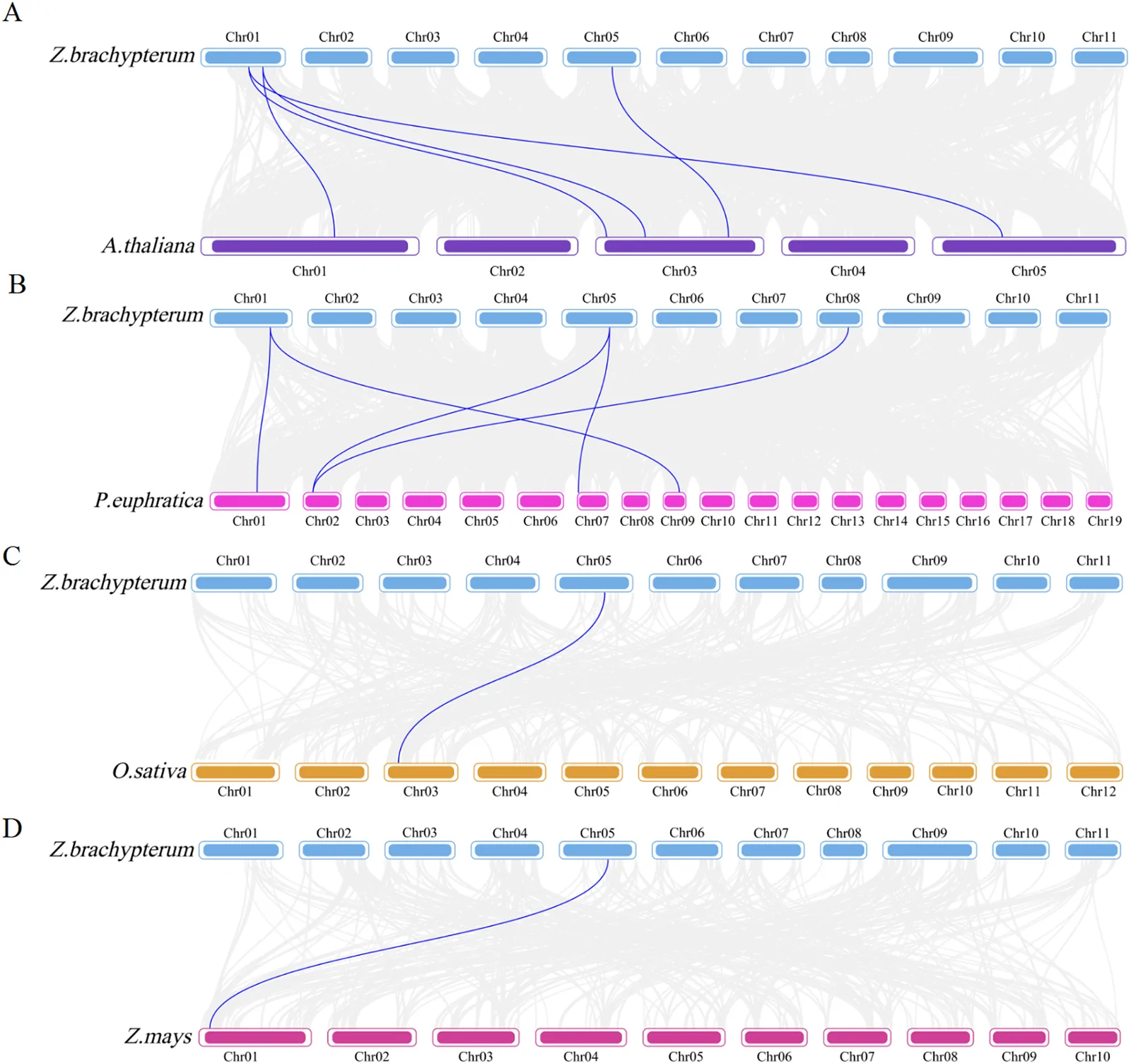
Genome-wide synteny analysis of BCAT gene loci between *Z. brachypterum* and four representative plant species. **(A)** Synteny between *Z. brachypterum* and *A. thaliana*; **(B)** Synteny between *Z. brachypterum* and *P. euphratica*; **(C)** Synteny between *Z. brachypterum* and *O. sativa*; **(D)** Synteny between *Z. brachypterum* and *Z. mays*.

### 3.5 Gene structure and conserved domain analysis of *ZbBCAT* gene family members

To explore structural features and functional differentiation of the ZbBCAT family, conserved motifs, functional domains, and exon–intron structures were systematically analyzed in the context of phylogenetic subfamily classification. Evolutionarily related members exhibited highly similar motif compositions and gene structure patterns, with clear subfamily specificity consistent with phylogenetic grouping (**Figure 4**). Conserved motif analysis showed that Zb8G000817, Zb8G000819, Zb5G001696, and Zb1G021424 all share core motifs Motif 1 to Motif 7, which form the catalytic backbone of BCAT proteins. Subfamily-specific motif variations were also observed: Zb8G000817 additionally contains Motif 8, 9, and 10; Zb8G000819 additionally contains Motif 8; Zb5G001696 and Zb1G021424 additionally contain Motif 9. By contrast, Zb1G011066 contains only Motif 4 and Motif 10, differing substantially from other members. Variation in motif number and composition reflects the structural diversity of the ZbBCAT family (**Figure 4A**). Functional domain validation via Pfam revealed distinct domain compositions across subfamilies. The BCAT1/2 and BCAT3 subfamilies all contain the canonical PLN02782 superfamily domain (branched-chain amino acid aminotransferase domain), consistent with their roles in core BCAA metabolism. In the BCAT4/5/6 subfamily, *Zb1G011066* harbors D-AAT_like and Sulfotransfer_5 superfamily domains, while *Zb1G021424* contains the PLN03117 superfamily domain, suggesting divergent substrate specificities and biological functions distinct from classical BCATs (**Figure 4B**). Gene structure analysis showed that *ZbBCAT* genes contain 9 to 19 exons. Members within the same subfamily share highly similar exon–intron numbers, distribution patterns, and length features, further supporting the reliability of subfamily classification (**Figure 4C**).

**Figure 4:**
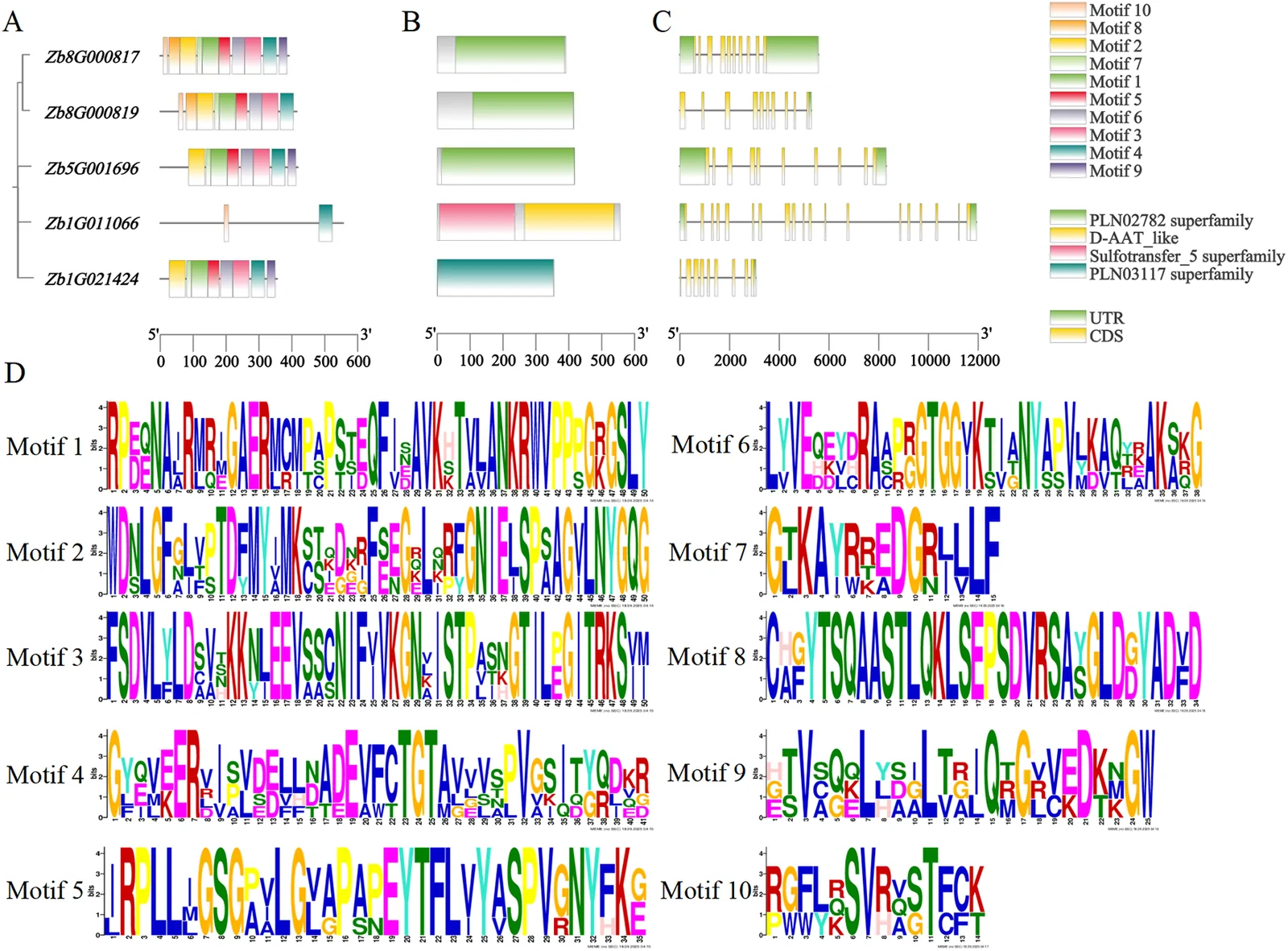
Analysis of gene structure and conserved structural domains of ZbBCATs. **(A)** Distribution of conserved domains in ZbBCAT. **(B)** Pfam model of ZbBCAT; green indicatesPLN02782 superfamily, yellow indicates D-AAT_like superfamily, red indicates Sulfotransfer_5 superfamily, and blue indicates PLN03117 superfamily. **(C)** Gene structure of *ZbBCAT*. Yellow boxes indicate exons, black lines indicate introns, and green indicates untranslated regions (UTRs). **(D)** Ten conserved motifs of ZbBCATs, with single-letter abbreviations representing amino acids.

### 3.6 Prediction of secondary and tertiary protein structures of ZbBCAT family members

Secondary structure prediction revealed that all ZbBCAT proteins consist of four structural elements, namely α-helix, β-turn, extended strand, and random coil (**Supplementary Table S4**). Random coil accounts for the highest proportion, followed by α-helix; together they form the core spatial scaffold that maintains conformational stability of the catalytic domain. β-turn and extended strand make up smaller proportions, functioning in local conformational fine-tuning and inter-element linkage. This secondary structure profile is typical of the aminotransferase family and provides the structural basis for functional specificity among family members.

Three-dimensional structural models of the five ZbBCAT proteins were generated via homology modeling using the SWISS-MODEL server, with global quality assessed by GMQE and QMEANDisCo Global metrics (**Supplementary Table S5**). For all models, the core catalytic domains showed high local structural confidence, whereas the flexible N-terminal transit peptides and C-terminal variable regions had lower confidence scores, consistent with the intrinsically disordered nature of these terminal segments. Zb1G021424 had the highest overall model quality (GMQE = 0.78, QMEANDisCo Global = 0.76 ± 0.05). Even Zb1G011066, which had the lowest global GMQE value (0.33), retained a reliably folded core functional domain. Three-dimensional structural models were further constructed via homology modeling. Tertiary structures showed subfamily specificity consistent with phylogenetic relationships (**Figure 5**). Both Zb8G000817 and Zb8G000819 contain a conserved core catalytic domain belonging to the Aminotran_4 superfamily. This domain forms the major folded functional region of mature BCAT proteins, excluding the N-terminal cleavable organelle transit peptide. Within this core domain, the key active site corresponds to a conserved catalytic lysine residue, which is responsible for covalent binding of the cofactor pyridoxal phosphate (PLP) and acts as the functional core of the BCAT active center. This catalytic lysine residue maintains a highly consistent spatial position in both protein models, reflecting the conserved spatial arrangement of the active site core. By contrast, the tertiary structures of Zb5G001696, Zb1G021424, and Zb1G011066 show varying degrees of divergence, with distinct conformations of core functional domains, suggesting functional specialization during long-term evolution. These structural evolutionary features provide structural biology insights into the molecular adaptation of the BCAT family to stress in *Z. brachypterum*.

**Figure 5:**
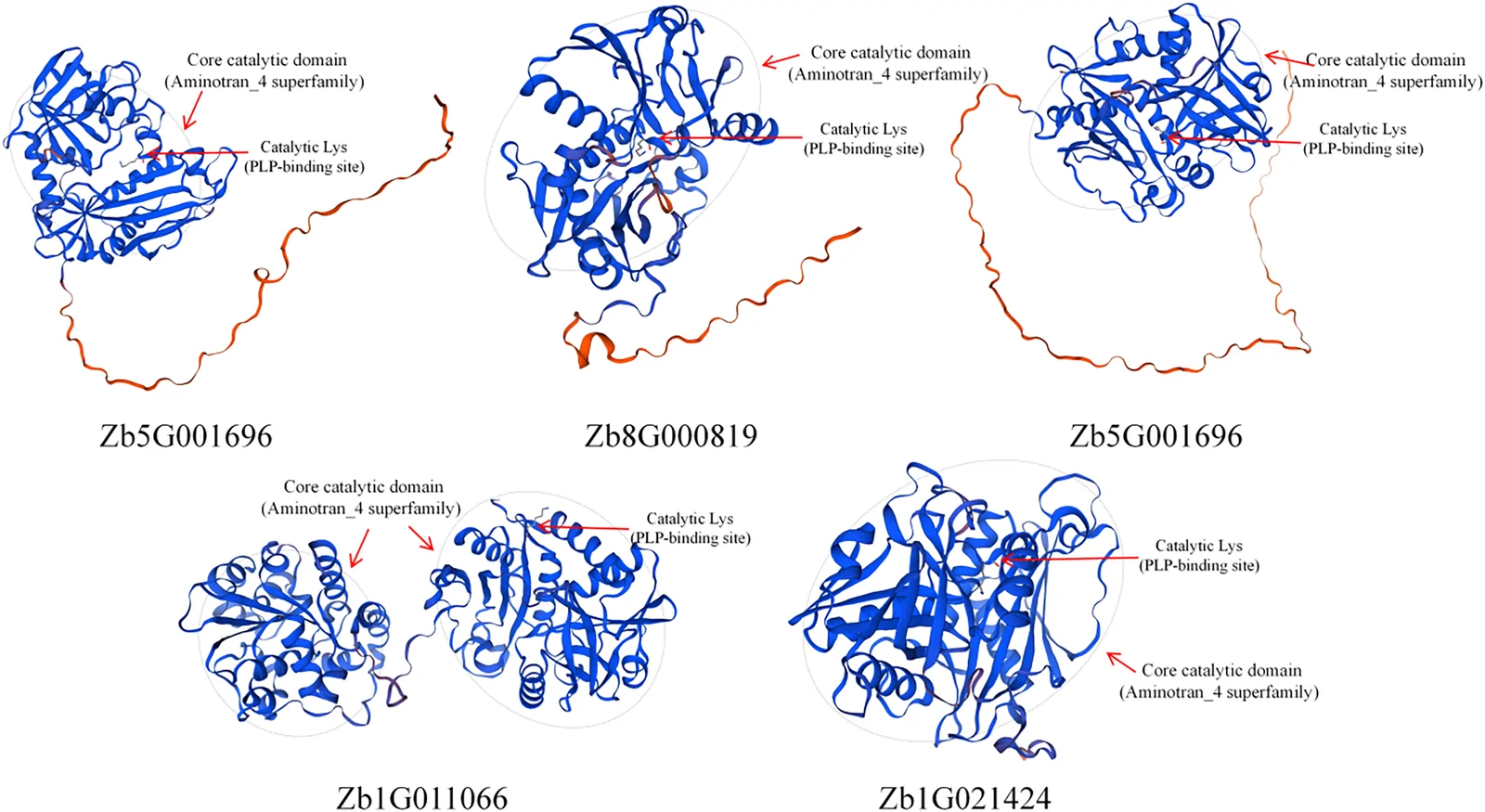
Tertiary structure homology models of five ZbBCAT family proteins from *Z. brachypterum.* All models were generated via the SWISS-MODEL homology modeling server. The color scale indicates the per-residue local structural quality evaluated by the QMEANDisCo score: dark blue = very high quality, light blue = good quality, yellow = moderate quality, orange = low quality.

### 3.7 Prediction of cis-acting elements in *ZbBCAT* gene promoters

To investigate the transcriptional regulatory mechanisms and potential functions of *ZbBCAT* genes, the 2000 bp promoter sequences upstream of the translation start site were extracted and analyzed for cis-acting elements using the PlantCARE database (**Figure 6**). A total of 152 cis-acting elements were identified across the five promoters, categorized into four functional classes: light-responsive elements, phytohormone-responsive elements, stress-responsive elements, and growth/development-related elements. Light-responsive elements were the most abundant among all predicted cis-acting elements, suggesting that the transcription of *ZbBCAT* genes may be broadly responsive to light signals (Maloney et al., 2010). Phytohormone-responsive elements include salicylic acid-responsive elements (TCA-element), gibberellin-responsive elements (TATC-box, GARE-motif), ABA-responsive elements (ABRE), and methyl jasmonate-responsive elements (TGACG-motif, CGTCA-motif), indicating that the family integrates multiple hormone signaling pathways to coordinate growth, development, and stress responses. Stress-responsive elements include defense/stress elements (TC-rich repeats, WUN-motif), drought-responsive elements (MBS), anaerobic response elements (ARE), and low-temperature responsive elements (LTR), suggesting that *ZbBCAT* genes perceive multiple abiotic stress signals and contribute to stress adaptation in *Z. brachypterum*. These elements provide a transcriptional basis for the differential expression of family genes under salt stress. Growth/development-related elements are relatively fewer, including auxin-responsive elements (AuxRR-core), seed-specific elements (RY-element), and endosperm expression elements (GCN4_motif), implying roles in seed development and tissue-specific growth. In summary, *ZbBCAT* promoters integrate light, hormone, and multiple stress signals to form a complex transcriptional regulatory network that governs both normal development and abiotic stress adaptation in *Z. brachypterum.*

**Figure 6:**
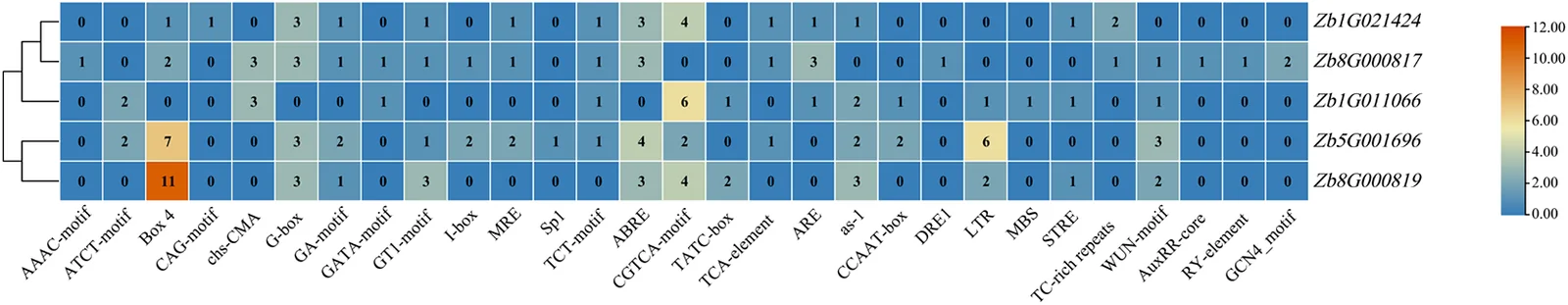
Distribution heatmap of cis-acting elements in ZbBCAT family promoters. Values indicate the copy number of each element; darker color represents a higher number of elements.

### 3.8 Spatiotemporal expression patterns of *ZbBCAT* genes under salt stress

To explore the response patterns of five candidate genes under salt stress, we performed quantitative real-time PCR (qRT-PCR) to analyze their transcript levels in leaves, stems and roots of *Z. brachypterum* treated with 500 mM NaCl for 0, 24, 48 and 72 h (**Figure 7**). The results showed that all genes exhibited obvious tissue-specific and time-course expression characteristics, indicating functional divergence among family members in salt stress adaptation.

**Figure 7:**
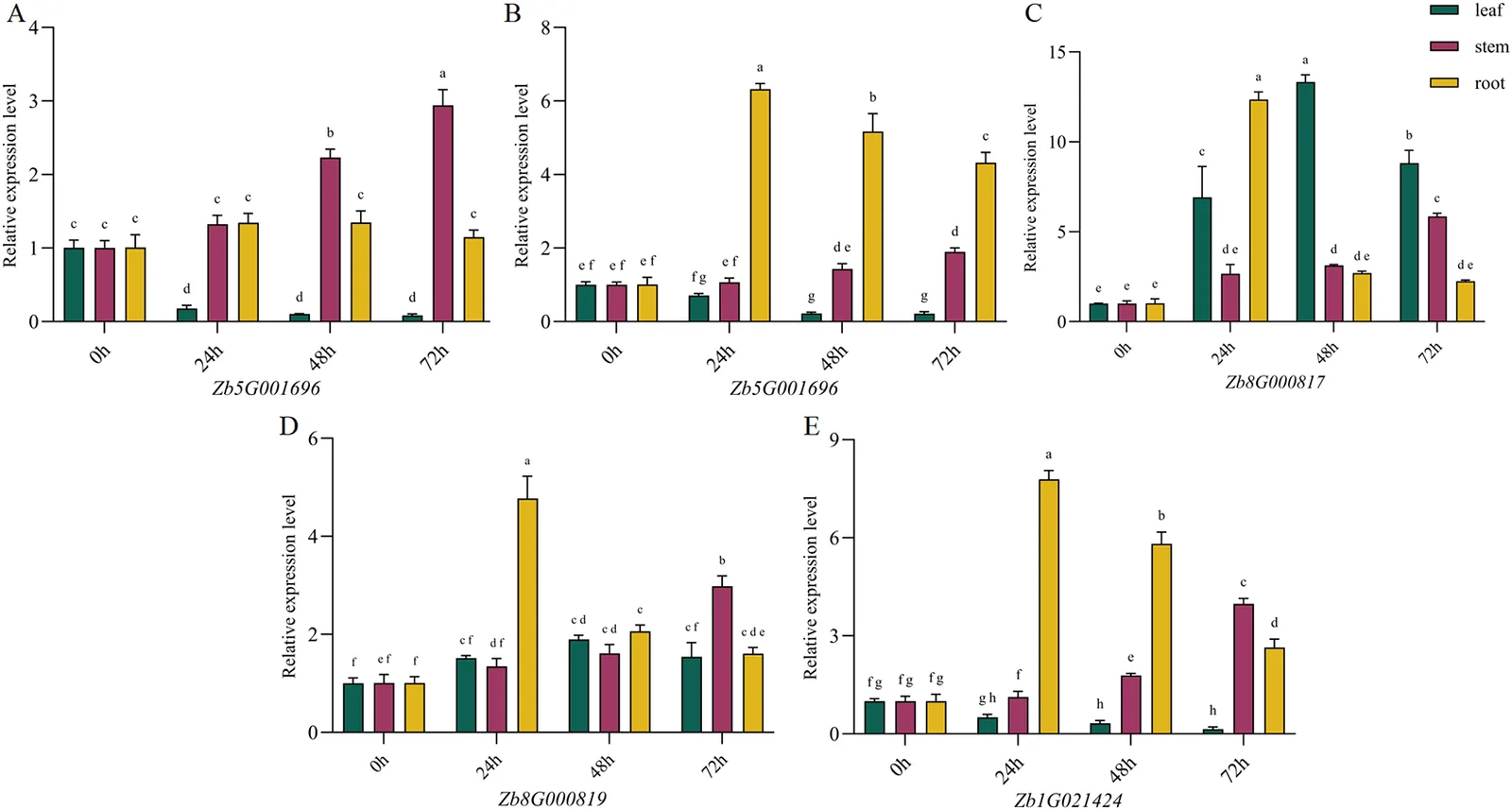
Expression profiles of five genes in different tissues under salt stress. **(A)** *Zb5G001696*; **(B)** *Zb1G011066*; **(C)** *Zb8G000817*; **(D)** *Zb8G000819*; **(E)** *Zb1G021424*. Relative transcript levels in leaves, stems and roots were determined by qRT-PCR after 500 mM NaCl treatment for 0, 24, 48 and 72 h. Data are presented as mean ± SD of three biological replicates. Different lowercase letters above bars indicate significant differences at *P* < 0.05 as determined by two-way analysis of variance followed by Tukey’s *post-hoc* test.

In leaves, *Zb8G000817* was the most strongly induced gene. Its expression increased continuously with the extension of stress time, reached the peak at 48 h (about 13-fold compared with the 0 h control), and then decreased slightly at 72 h while still maintaining a high level. *Zb8G000819* showed a mild upregulation trend, with the maximum expression at 48 h. By contrast, *Zb5G001696*, *Zb1G011066* and *Zb1G021424* were generally downregulated under salt stress; their transcript levels decreased significantly from 24 h and remained at low levels thereafter.

In stems, all five genes were upregulated to different degrees, but their peak expression times varied. *Zb8G000817* showed the strongest induction, with its expression rising steadily and peaking at 72 h. *Zb5G001696*, *Zb8G000819* and *Zb1G021424* shared similar continuous upregulation trends, all reaching the highest expression at 72 h. *Zb1G011066* presented a relatively delayed response, with no obvious change in the early stage of stress, and only moderate upregulation at 72 h.

In roots, the genes generally responded to salt stress rapidly. *Zb8G000817* was significantly induced at 24 h, reached the peak at 48 h, and then decreased markedly at 72 h. *Zb8G000819* and *Zb1G021424* showed similar transient induction patterns, with expression peaking at 24 h followed by a gradual decline. *Zb1G011066* was notably upregulated at 48 h and then decreased at 72 h. *Zb5G001696* exhibited a relatively weak response, with only slight upregulation during the whole treatment period.

Collectively, *Zb8G000817* (*ZbBCAT2*) was the only gene significantly and stably induced by salt stress in all three tissues, with the highest upregulation amplitude. Therefore, *Zb8G000817* was selected as the core candidate for subsequent functional validation.

### 3.9 Subcellular localization of ZbBCAT2 in *N. benthamiana*

To determine the subcellular distribution of ZbBCAT2 in planta, we performed transient expression assays in *N. benthamiana* leaf epidermal cells. The 35S::*ZbBCAT2*-YFP fusion construct was delivered via Agrobacterium-mediated infiltration, with the 35S::YFP empty vector serving as the negative control. Chlorophyll autofluorescence was adopted as an endogenous marker for chloroplasts, and fluorescence signals were captured by confocal laser scanning microscopy. As shown in **Figure 8**, the 35S::YFP control exhibited a diffuse fluorescence pattern throughout the cytoplasm and nucleus, with no detectable co-localization with chlorophyll autofluorescence. In contrast, the YFP signal from 35S::*ZbBCAT2*-YFP presented as distinct punctate foci that highly overlapped with the red chlorophyll autofluorescence. These results confirm that ZbBCAT2 is a chloroplast-targeted protein in plant cells.

**Figure 8:**
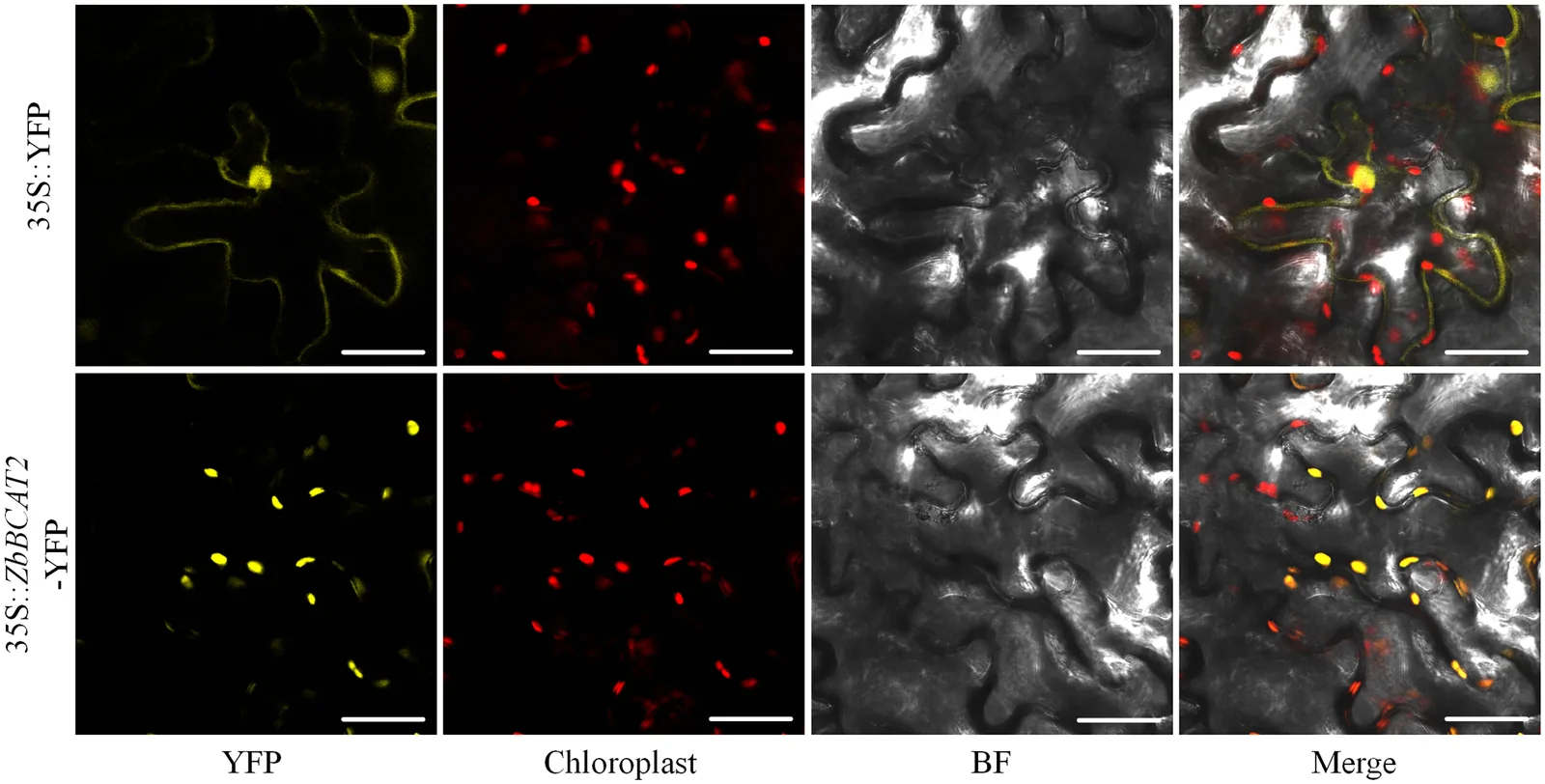
Subcellular localization analysis of ZbBCAT2 in *N. benthamiana* leaf epidermal cells. The upper row presents the localization of 35S::YFP as the negative control. The lower row presents the localization of the 35S::*ZbBCAT2*-YFP fusion protein. For each row, images from left to right correspond to the YFP fluorescence channel, the chloroplast channel highlighted by endogenous chlorophyll autofluorescence, the bright field channel showing epidermal cell morphology, and the merged image of all three channels. Scale bar = 30 μm.

### 3.10 Preliminary functional validation of *ZbBCAT2* in *A. thaliana*

To verify the biological function of *ZbBCAT2* (*Zb8G000817*) in salt stress response, a plant overexpression vector was constructed and transformed into *A. thaliana* via the floral dip method. T_3_ homozygous transgenic lines were obtained after multi-generation selection. qRT-PCR confirmed that *ZbBCAT2* had no basal expression in wild-type Col-0 or the *bcat2* loss-of-function mutant. In three overexpression lines (OE6, OE27, OE8), target gene expression was 7–13-fold higher than in the wild type, verifying successful heterologous expression for subsequent phenotypic assays (**Figure 9B**). Four-week-old seedlings of wild-type Col-0, *bcat2* mutant, and overexpression lines were subjected to continuous 500 mM NaCl stress for 3 weeks. Under normal conditions, no significant growth differences were observed among genotypes. After salt stress, wild-type plants showed obvious leaf chlorosis and albinism; the *bcat2* mutant had only mild chlorosis and the least damage; overexpression lines exhibited severe leaf bleaching and partial plant death (**Figure 9A**). Statistical analysis showed a Leaf bleaching rate of 22.23% in wild-type Col-0, compared with only 4.91% in the *bcat2* mutant. All three overexpression lines had leaf bleaching rates above 30.47%, significantly higher than the wild type (**Figure 9C**). These results indicate that *ZbBCAT2* overexpression exacerbates salt stress damage in *A. thaliana* seedlings and negatively regulates salt tolerance.

**Figure 9:**
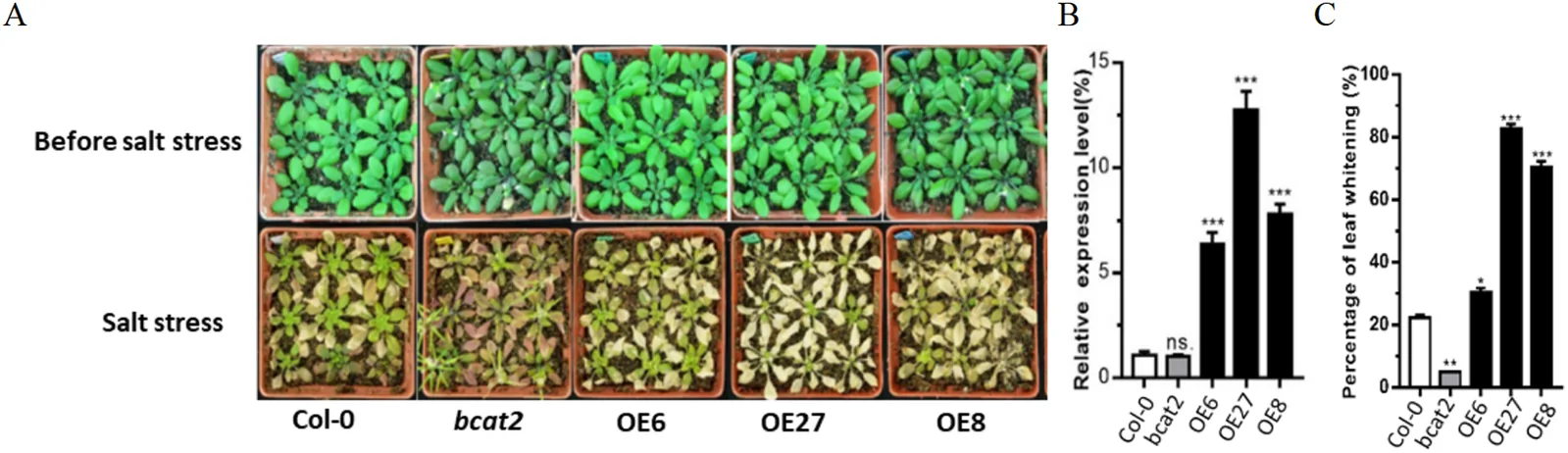
Phenotypic analysis of salt tolerance in *ZbBCAT2* transgenic Arabidopsis lines. **(A)** Growth phenotypes of wild-type Col-0, *bcat2* mutant and three *ZbBCAT2* overexpression lines (OE6, OE27, OE8) before and after salt stress treatment. **(B)** qRT-PCR verification of *ZbBCAT2* transcript levels in the indicated lines. **(C)** Quantitative statistics of leaf whitening percentage in each line following salt stress. Data are presented as mean ± SD of three biological replicates. *, ** and *** indicate significant differences at *P* < 0.05, *P* < 0.01 and *P* < 0.001, respectively; ns indicates no significant difference compared with Col-0.

### 3.11 Preliminary functional validation of *ZbBCAT2* in *O. sativa*

To test evolutionary conservation in monocots, the same overexpression construct was transformed into ZH11via Agrobacterium-mediated callus transformation, and homozygous transgenic lines were obtained. qRT-PCR showed 2–3-fold higher *ZbBCAT2* expression in OE1 and OE7 lines than in wild-type ZH11(**Figure 10**). Two-leaf-stage seedlings were treated with 500 mM NaCl for 2 weeks. Wild-type plants retained partial green leaves, whereas overexpression lines showed extensive leaf yellowing and wilting, with significantly more severe damage. Survival rates of overexpression lines were extremely significantly lower than that of the wild type. This result is consistent with the findings in *A. thaliana*, demonstrating that *ZbBCAT2* exerts consistent negative regulatory effects on salt tolerance when heterologously expressed in both dicot and monocot hosts.

**Figure 10:**
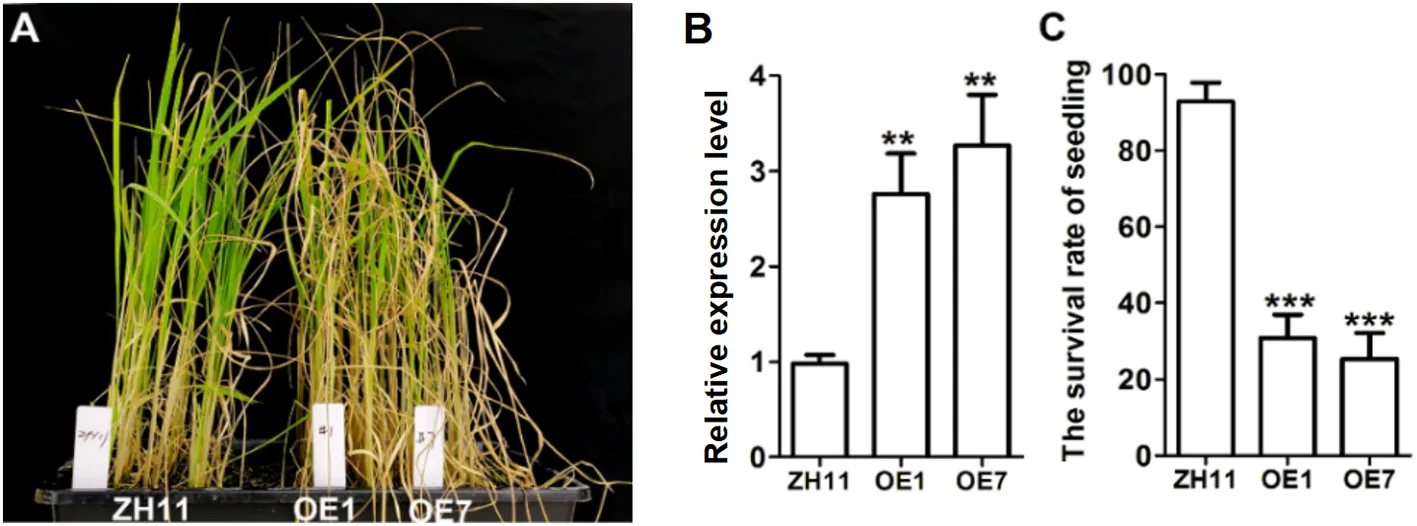
Salt tolerance analysis of *ZbBCAT2*-overexpressing *O. sativa* lines. **(A)** Growth phenotypes of wild-type ZH11 and two *ZbBCAT2* overexpression lines (OE1 and OE7) after salt stress treatment. **(B)** qRT-PCR verification of *ZbBCAT2* transcript levels in the indicated lines. **(C)** Statistical analysis of seedling survival rate in each line after salt stress. Data are presented as mean ± SD of three biological replicates. ** and *** indicate significant differences at *P* < 0.01 and *P* < 0.001, respectively, compared with the wild-type ZH11.

## 4 Discussion

### 4.1 Evolutionary conservation and functional differentiation of the *ZbBCAT* gene family

BCATs are core enzymes regulating BCAA metabolic homeostasis in plants, with pivotal roles in growth, development, and abiotic stress responses. The transamination they catalyze mediates both BCAA biosynthesis and catabolism, two opposing processes (Diebold et al., 2002; Binder, 2010). In this study, we identified five *ZbBCAT* genes in *Z. brachypterum* at the genome-wide scale for the first time (**Table 1**). The family size is comparable to *O. sativa* (five members) and slightly smaller than *A. thaliana* and *S. lycopersicum* (Diebold et al., 2002; Kochevenko et al., 2012; Jin et al., 2019), suggesting overall evolutionary conservation of the BCAT family in higher plants, with copy numbers maintained within a stable range that reflects its functional importance in basal metabolism. Phylogenetic analysis divided the five *ZbBCATs* into three subfamilies with distinct copy number evolution patterns between monocots and dicots (**Figure 2**). The BCAT3 subfamily is the largest and has expanded in monocots via segmental duplication, likely resulting in functional redundancy, whereas it remains single-copy in dicots with highly conserved basal synthetic functions (Schuster and Binder, 2005; Jin et al., 2019). This pattern of monocot expansion and dicot conservation is typical of plant primary metabolism gene families: core metabolic enzymes often acquire redundant copies in monocots, while dicots retain single copies for precise pathway regulation (Bekaert et al., 2011; Li et al., 2016). The BCAT1/2 subfamily is dicot-specific, with no orthologs in *O. sativa* or *Z. mays*. A species-specific tandem duplication gave rise to *Zb8G000817* and *Zb8G000819* in *Z. brachypterum*, whereas only one copy exists in *P. euphratica*. Tandem duplication of stress-related metabolic genes is prevalent in desert plants, enhancing pathway response flexibility via dosage effects as an adaptive strategy to extreme arid and saline environments (Mohanta et al., 2024). Duplication is typically followed by subfunctionalization or neofunctionalization, driving functional diversity (Flagel and Wendel, 2009); this duplication event may therefore be linked to the adaptation of *Z. brachypterum* to harsh desert conditions. The BCAT4/5/6 subfamily has contracted in *Z. brachypterum*, *P. euphratica*, and *Z. mays*, retaining only a few copies. This indicates functional specialization and streamlining, with some secondary metabolic roles potentially replaced by other gene families, consistent with the evolutionary “use-it-or-lose-it” principle (Mira et al., 2001; Moghe and Last, 2015). Multi-species synteny analysis supports these evolutionary inferences: five pairs of syntenic BCAT homologs exist between *Z. brachypterum* and each dicot species, preserving an intact syntenic backbone; only one syntenic pair (BCAT3) is shared with monocots (**Figure 3**). This confirms profound evolutionary divergence of the BCAT family between monocots and dicots, with loss of synteny in other subfamilies providing the substrate for functional diversification (Panchy et al., 2016).

### 4.2 Gene structure, conserved motifs and promoter cis-element analysis of the *ZbBCAT* gene family

Gene structure and motif analyses revealed high congruence with phylogenetic subfamily classification (**Figures 4A, B**). The BCAT1/2 and BCAT3 subfamilies both possess the complete PLN02782 BCAT domain and share core catalytic motifs 1–7, including conserved pyridoxal phosphate-binding sites and catalytic centers, which preserve fundamental aminotransferase function (Okada et al., 1997; Binder, 2010). High conservation of core catalytic residues also supports a monophyletic origin of the BCAT family (Yennawar et al., 2001). The BCAT4/5/6 subfamily shows greater structural divergence, with *Zb1G011066* harboring D-AAT_like and sulfotransferase domains, implying deviation from classical BCAT substrate specificity and involvement in specialized secondary metabolism. This structural subfamily differentiation underlies the functional diversity of the family. Promoter cis-element analysis provides transcriptional insights into stress responsiveness (**Figure 6**). The 152 identified elements span light, hormone, stress, and developmental categories. Light-responsive elements are most abundant, consistent with *BCAT* functions in photosynthetic tissue amino acid metabolism (Binder, 2010). Importantly, promoters contain widespread ABRE, MBS, LTR, and TC-rich repeats, as well as salicylic acid and methyl jasmonate elements, enabling integration of multiple stress and hormone signals during stress adaptation. The prevalence of ABRE elements aligns with the reported role of *AtBCAT2* in ABA-dependent BCAA metabolism (Huang and Jander, 2017). ABA-responsive bZIP transcription factors can directly bind ABRE motifs to regulate *BCAT* expression under stress, a pathway highly conserved in dicots (Pedrotti et al., 2018), which explains the salt-induced expression changes of *ZbBCAT* genes. Cis-element divergence among family members also drives tissue-specific expression and differential stress responsiveness (Wittkopp and Kalay, 2011).

### 4.3 Functional differentiation and key gene selection reflected by tissue-specific expression

The tissue-specific expression patterns of *ZbBCAT* family members may reflect extensive functional divergence in salt stress adaptation, and also may provide a rational basis for screening core functional genes (**Figure 7**). As the first organ directly exposed to salt stress, roots act as the frontline of plant stress perception and defense, and the widespread rapid induction of *ZbBCAT* genes in roots suggests that BCAA metabolic reprogramming may represents a critical early adaptive response in root tissue (Zhao et al., 2014; Batista-Silva et al., 2019). Among these genes, *Zb8G000817* displays the earliest and strongest induction, implying that it may function as the core isoform involved in regulating BCAA homeostasis in salt-stressed roots, and may contribute to root osmotic adjustment and oxidative damage repair by modulating BCAA accumulation. In contrast, *Zb1G021424*, *Zb8G000819* and *Zb5G001696* only exhibit transient or weak upregulation in roots followed by gradual decline, suggesting that these members may be mainly involved in basal BCAA metabolism under normal conditions and may be insufficient to meet the sustained metabolic requirements of roots under prolonged salt stress. In leaf tissue, the specific and sustained upregulation of *Zb8G000817* is particularly notable: given that *de novo* BCAA biosynthesis in plants is predominantly localized in chloroplasts (Galili et al., 2016), this expression pattern suggests that *Zb8G000817* may act as a key isozyme maintaining BCAA homeostasis in photosynthetic tissues against the osmotic and oxidative damage induced by salt stress, whereas other family members appear to be primarily involved in basal metabolism and thus show overall downregulation in leaves after stress treatment. The staggered peak expression of *ZbBCAT* genes in stems is consistent with the role of stems as the transport hub connecting source and sink organs, which may reflect the dynamic regulation of BCAA long-distance transport and local metabolic turnover under sustained stress. Notably, the marked difference in stress induction amplitude between the tandem duplicated pair *Zb8G000817* and *Zb8G000819* suggests that cis-regulatory element divergence in promoter regions, rather than coding sequence variation, may drive subfunctionalization of this lineage after duplication, with *Zb8G000817* having evolved into the dominant stress-responsive isoform functioning across multiple tissues. Overall, as the only member consistently and strongly induced in all three tested tissues with the highest upregulation amplitude, *Zb8G000817* (*ZbBCAT2*),which is the putative ortholog of stress-responsive *AtBCAT2*, represents a promising candidate gene mediating salt tolerance in *Z. brachypterum*, laying a foundation for subsequent functional validation and dissection of stress resistance mechanisms.

### 4.4 Metabolic mechanism underlying negative regulation of salt tolerance by *ZbBCAT2*

Heterologous overexpression in both *A. thaliana* and *O. sativa* demonstrated that *ZbBCAT2* negatively regulates salt tolerance (**Figures 9**, **10**). Under 500 mM NaCl, overexpression lines showed increased leaf damage and reduced survival, while the *bcat2* mutant showed enhanced tolerance. This consistent phenotypic performance across dicot and monocot heterologous expression systems reflects that the chloroplastic biosynthetic activity of *ZbBCAT2* is biochemically conserved enough to produce equivalent metabolic flux perturbations in divergent plant hosts. Notably, this does not mean that the endogenous salt tolerance function of native *BCAT2* orthologs is evolutionarily conserved across angiosperms; instead, endogenous *BCAT* family members have undergone marked lineage-specific functional divergence, as detailed below. Based on subcellular localization analysis and orthologous function conservation, we propose a “substrate shunting – coenzyme A deficiency” working hypothesis to explain the potential underlying molecular mechanism. ZbBCAT2 localizes to chloroplasts (**Figure 8**), and by homology with its biochemically characterized ortholog AtBCAT2, it likely functions as a biosynthetic isozyme catalyzing BCAA production from branched-chain keto acids such as 3-methyl-2-oxobutanoate (Diebold et al., 2002; Huang and Jander, 2017). 3-methyl-2-oxobutanoate (α-ketoisovalerate) is a shared branching precursor of valine synthesis and pantothenate (vitamin B5)/coenzyme A (CoA) synthesis, with competing metabolic flux in chloroplasts (Leonardi and Jackowski, 2007). As a key branch point, its partitioning directly controls the balance between BCAA and CoA biosynthesis (Sun et al., 2024). We hypothesize that salt-induced upregulation of *ZbBCAT2* shunts more 3-methyl-2-oxobutanoate toward BCAA synthesis, thereby reducing substrate availability for CoA production — an essential cofactor for energy metabolism, biosynthesis, and antioxidant defense (Falk and Guerra, 1993; Hanson et al., 2016). Salt stress increases cellular demand for energy and redox homeostasis; insufficient CoA reduces tricarboxylic acid (TCA) cycle flux and ATP production. Genetic evidence from *A. thaliana* confirms that *PPAT*, a key CoA biosynthetic gene, determines cellular CoA levels and is a limiting factor for salt tolerance (Rubio et al., 2008). The TCA cycle is the main source of reducing power under stress. Reduced TCA flux impairs glutathione regeneration and reactive oxygen species (ROS) scavenging. Multi-omics studies in alfalfa show that TCA-derived NADPH fuels glutathione redox cycling; reduced TCA activity disrupts redox homeostasis (Li et al., 2020), leading to enhanced membrane lipid peroxidation and chlorophyll degradation. CoA deficiency also impairs chloroplast fatty acid synthesis and redox balance, further compromising photosystem stability and creating a vicious cycle of metabolic damage. In line with this hypothesis, our previous transcriptome data from salt-stressed Zygophyllum revealed that samples with upregulated BCAA synthesis exhibited coordinated downregulation of pantothenate–CoA and TCA cycle genes, with a significant negative correlation between the two biological processes (Wang et al., 2020). Collectively, the core biochemical logic of this regulatory pathway, which consists of substrate shunting, coenzyme A deficiency and oxidative damage, is partially conserved across plant lineages, whereas endogenous BCAT family genes in distinct plant groups have evolved lineage-specific functions and regulatory patterns.

### 4.5 Evolutionary mechanism of functional divergence between monocot and dicot BCATs

The negative salt tolerance function of *ZbBCAT2* contrasts with the reported positive role of *OsBCAT2* (Sun et al., 2024). This is not an experimental discrepancy but a classic example of subfunctionalization during monocot–dicot BCAT family evolution: the two are paralogs from distinct evolutionary branches. Since their divergence approximately 140 million years ago, primary metabolic pathways have undergone independent evolutionary remodeling, and BCAT family diversification exemplifies this process (Chaw et al., 2004). Evolutionarily, *OsBCAT2* belongs to the monocot-specific BCAT3 subfamily and is more closely related to dicot BCAT3 genes. *ZbBCAT2* belongs to the dicot-specific BCAT1/2 subfamily, for which no monocot orthologs exist. The two diverged early in land plant evolution, with sufficient sequence divergence to enable functional differentiation. Biochemically, OsBCAT2 is a mitochondrion-localized catabolic isozyme that deaminates BCAAs to supply keto acid precursors for vitamin B5 and CoA synthesis, thereby positively regulating salt tolerance (Sun et al., 2024). ZbBCAT2 is a chloroplast-localized biosynthetic isozyme that produces BCAAs at the expense of CoA precursor pools, exerting negative regulation. Their different subcellular localizations and opposing catalytic directions produce opposite effects on the same metabolic node, resulting in divergent overexpression phenotypes. Notably, functional divergence within the same dicot BCAT1/2 subfamily is also observed across species. AtBCAT2 has been confirmed to localize to mitochondria and function as a catabolic isozyme in Arabidopsis (Angelovici et al., 2014), whereas ZbBCAT2 in our study shows chloroplast targeting and a biosynthetic role in leaf tissues. This pattern suggests that subfunctionalization of the BCAT1/2 subfamily has likely occurred independently in different dicot lineages, further reflecting the high functional flexibility of the BCAT family. This functional differentiation is not unique to the monocot–dicot divide but is a general feature of plant BCAT evolution. Within a single species, BCATs typically partition biosynthetic and catabolic functions via subcellular localization: in *A. thaliana*, mitochondrial AtBCAT1 initiates BCAA degradation (Schuster and Binder, 2005), while chloroplastic AtBCAT2 mediates biosynthesis (Diebold et al., 2002), acting in opposition to maintain homeostasis. Functional specialization also varies among dicot species. In *S. lycopersicum*, chloroplastic SlBCAT3/4 are biosynthetic, while mitochondrial SlBCAT1/2 are catabolic; during fruit development, catabolic BCAT activity predominates to supply respiratory carbon (Kochevenko and Fernie, 2011). The BCAT family thus exhibits high functional flexibility during plant adaptive evolution, with lineage diversification via gene duplication, subcellular partitioning, and tissue-specific expression. The functional divergence between ZbBCAT2 and OsBCAT2 reflects this evolutionary flexibility and highlights distinct metabolic strategies for salt adaptation in monocots versus dicots.

### 4.6 Conclusion

In this study, we perform the first genome-wide identification and bioinformatic characterization of the BCAT gene family in the desert halophyte *Z. brachypterum*, and conduct preliminary functional exploration of the core stress-responsive member *ZbBCAT2* in the context of salt stress response.

The main findings and inferences drawn from this work are as follows. First, five *ZbBCAT* genes identified across the genome can be phylogenetically classified into three evolutionary subfamilies, with apparent copy number variation that may underlie functional divergence between monocot and dicot lineages. *ZbBCAT2* belongs to the dicot-specific BCAT1/2 subfamily, and the tandem-duplicated paralog pair *Zb8G000817/Zb8G000819* appears to have evolved under strong purifying selection following the duplication event. Second, gene structures and conserved motif compositions show high congruence with phylogenetic subfamily grouping. Promoter regions are enriched with light-, phytohormone- and stress-responsive cis-acting elements, which may provide a transcriptional regulatory basis for the differential salt responsiveness observed for *ZbBCAT* genes. Third, under salt stress, *ZbBCAT* genes exhibit distinct tissue-specific expression patterns. *ZbBCAT2* is the only family member consistently and robustly upregulated in roots, stems and leaves, suggesting it may serve as the dominant stress-responsive isoform in this species. Fourth, heterologous overexpression of *ZbBCAT2* in both *A. thaliana* and *O. sativa* appears to aggravate salt stress damage and reduce plant survival, whereas the *A. thaliana bcat2* loss-of-function mutant shows enhanced salt tolerance. These phenotypic results collectively suggest that ZbBCAT2 may function as a negative regulator of salt tolerance. We tentatively propose a “substrate shunting–coenzyme A deficiency” hypothesis to explain this potential effect: upregulation of *ZbBCAT2* may divert shared metabolic precursors from coenzyme A biosynthesis toward branched-chain amino acid synthesis, which could in turn impair tricarboxylic acid cycle flux and redox homeostasis under salt stress. Fifth, the functional discrepancy between ZbBCAT2 and the previously reported positive salt tolerance regulator OsBCAT2 may be attributed to deep evolutionary divergence of the *BCAT* family between monocots and dicots; the two proteins belong to distinct subfamilies with presumably different subcellular localizations and opposing catalytic preferences.

Collectively, this study provides a systematic characterization of the *BCAT* gene family in *Z. brachypterum* and presents preliminary evidence for a negative regulatory role of chloroplastic ZbBCAT2 in salt tolerance. Our findings offer tentative insights into BCAA-centered metabolic adaptation to salt stress in desert halophytes, and establish candidate gene targets and a working mechanistic hypothesis for follow-up functional and mechanistic studies.

### 4.7 Limitations and future perspectives

This study provides a systematic genome-wide characterization of the BCAT gene family in *Z. brachypterum* and preliminary evidence supporting that *ZbBCAT2* acts as a negative regulator of salt tolerance based on heterologous overexpression assays. Nevertheless, several important limitations should be noted. First, although the chloroplast localization of ZbBCAT2 has been experimentally confirmed using YFP fusion protein (**Figure 8**), its biosynthetic enzymatic activity remains inferred from bioinformatic homology and has not been verified by *in vitro* or *in-vivo* enzyme activity assays. Second, the substrate shunting-coenzyme A deficit model presented in this work represents only a working mechanistic hypothesis, which still lacks empirical support from targeted metabolomics and metabolic-flux tracing measurements of endogenous metabolites. Third, all functional conclusions are derived from heterologous overexpression in Arabidopsis and rice; the endogenous biological role of *ZbBCAT2* in native *Z. brachypterum* remains to be validated using gene-knockout or knockdown genetic materials.

Future work will address these limitations along three main research directions. CRISPR-Cas9-mediated knockout mutants of *ZbBCAT2* will be generated in *Z. brachypterum*, and biochemical function will be further characterized by enzyme-activity measurements. Widely-targeted metabolomics combined with stable-isotope tracing will be applied to test the proposed BCAA-CoA-related metabolic changes and resolve the precise molecular mechanism underlying salt-stress responses. Furthermore, upstream transcription factors and downstream interacting partners of *ZbBCAT2* will be screened to reconstruct its regulatory network. Collectively, this work identifies a promising gene candidate for salt-stress research in desert halophytes and offers new insights into functional divergence within the plant BCAT family.

## Data Availability

Publicly available datasets used in this study include: *Arabidopsis thaliana BCATs* reference sequences from TAIR: https://www.arabidopsis.org/; Genome and *BCATs* gene sequences of *Oryza sativa*, *Zea mays* and *Populus euphratica* from NCBI GenBank/RefSeq: https://www.ncbi.nlm.nih.gov/; Conserved domain profiles from Pfam (https://www.ebi.ac.uk/interpro/) and NCBI CDD; Promoter cis-element data from PlantCARE: https://bioinformatics.psb.ugent.be/webtools/plantcare/html/; and Protein structure templates from SWISS-MODEL: https://swissmodel.expasy.org/. The reference genome of *Zygophyllum brachypterum* is an in-house assembled dataset generated by our laboratory and has not yet been publicly released. It is available from the corresponding author upon reasonable request. Requests to access these datasets should be directed to Peipei Jiao, jiaopeipei2000@126.com.
