# Supplementary material for: Genome-wide identification of the BCAT gene family in Zygophyllum brachypterum and functional characterization of ZbBCAT2 in salt stress response
Source: Front Plant Sci. 2026 Oct 19;17:1978750. doi: 10.3389/fpls.2026.1978750 (PMC13725701; doi:10.3389/fpls.2026.1978750)
Supplement: Supplementary file 1 [file DataSheet1.docx]

Supplementary Material

# 1 Supplementary Data

Table S1: Primer Sequences for PCR.

| Primer Name | Forward primer (5′→3′) | Reverse primer (5′→3′) | Amplification efficiency (%) | R² |
| --- | --- | --- | --- | --- |
| *q-Zb8G000819* | GTATGACCGTGCTGCTCGTAG | CTGGTGATTCCTTCAAGTATGG | 96.4 | 0.991 |
| *q-Zb8G000817* | TGCTTCTGGCTTGGACGACTA | GCCGACGGGCTAATCTCAATA | 95.8 | 0.989 |
| *q-Zb5G001696* | GAGGTTTAATCAGGCAGTC | TGAAATCAGATAATGTGGC | 97.2 | 0.993 |
| *q-Zb1G011066* | ACGGAGATGCTCAAATGGG | ACGGGAATGGCTTGGGATA | 93.7 | 0.986 |
| *q-Zb1G021424* | TTCTCGGATGTCCTGTTTC | AACCTCCTCAATGTTCTTCTT | 98.1 | 0.995 |
| *ZbActin* | TAGATGGCTGGAACAGAACTT | GTTGCTATTGATTATGAGAAGG | 99.3 | 0.994 |
| *At-Actin* | CGGAAAGCAGTTGGAGGATGG | CGGAGCCTGAGAACAAGATGAAG | 98.6 | 0.997 |
| *Os-Actin* | GGCGTTATGGTTGGGATGG | GCTCGGCAGAGGTGGTGAA | 97.8 | 0.994 |
| *Hind Ⅲ-ZbBCAT2* | AGAACACGATCGATAAGCTTATG  ACTTCAAGAATCAATTTTTCTAGA | GTCGCATGATCCATAAGCTTATCT  ATCACCACAACCCAAC | / | / |
| *SALK_037854* | CTCTTCCACCTTCTTTTTCGG | AAATCAAGAAACATTTGGGGG | / | / |
| *LBb1.3* | ATTTTGCCGATTTCGGAAC |  | / | / |
| *BCAT2* | GAGGCGTCAAGAGCATCACA | ACGAGGACTGAACGCAGTTT | / | / |

Table S2: Detailed chromosomal coordinates of *ZbBCAT* gene family members in *Zygophyllum brachypterum*.

| **Gene ID** | **Chromosome** | **Start (bp)** | **End (bp)** | **Strand** |
| --- | --- | --- | --- | --- |
| *Zb8G000817* | chr08 | 18134888 | 18140461 | + |
| *Zb8G000819* | chr08 | 18108660 | 18113947 | + |
| *Zb5G001696* | chr05 | 27592210 | 27600508 | + |
| *Zb1G011066* | chr01 | 26980714 | 26992642 | + |
| *Zb1G021424* | chr01 | 34865385 | 34868442 | + |

Table S3: Ka, Ks values and Ka/Ks ratio of the tandem-duplicated *ZbBCAT* paralog pair.

| **Gene_1** | **Gene_2** | **Ka** | **Ks** | **Ka/Ks** |
| --- | --- | --- | --- | --- |
| *Zb8G000817* | *Zb8G000819* | 0.099 | 0.218 | 0.454 |

Table S4: Prediction of Secondary Structures of ZbBCAT Family Members.

| **Gene ID** | **Percentage（%）** | | | | **DistributionofSecondaryStructureElements** |
| --- | --- | --- | --- | --- | --- |
|  | **Hh** | **Ee** | **Tt** | **Cc** |  |
| Zb8G000817` | 27.18 | 15.90 | 6.15 | 50.77 |  |
| Zb8G000819 | 29.71 | 17.63 | 5.56 | 47.10 |  |
| Zb5G001696 | 23.74 | 13.91 | 5.76 | 56.59 |  |
| Zb1G011066 | 38.02 | 15.32 | 5.59 | 41.08 |  |
| Zb1G021424 | 28.25 | 18.36 | 7.06 | 46.33 |  |

Table S5: Quality parameters of SWISS-MODEL models for five ZbBCAT proteins.

| **GeneName** | Zb8G000817 | Zb8G000819 | Zb5G001696 | Zb1G011066 | Zb1G021424 |
| --- | --- | --- | --- | --- | --- |
| **GMQE** | 0.67 | 0.58 | 0.63 | 0.33 | 0.78 |
| **QMEANDisCo Global** | 0.73 ± 0.05 | 0.70 ± 0.05 | 0.72 ± 0.05 | 0.59 ± 0.05 | 0.76 ± 0.05 |

# 2 Supplementary Figure

Figure S1. Validation of the *bcat2* T-DNA insertion mutant (SALK_037854). (A) Genotyping of the homozygous mutant by three-primer genomic PCR. Wild-type Col-0 shows a single amplicon corresponding to the intact endogenous allele, while the homozygous *bcat2* mutant yields only the T-DNA-flanking amplicon with no detectable wild-type product. (B) Semi-quantitative RT-PCR verification of endogenous *AtBCAT2* transcriptional silencing. AtActin was amplified as an internal reference to confirm equal cDNA loading across samples.
